# Comparative Analysis of Intraocular Lens Power Calculation Formulas (Kane, Barrett Universal II, Hill–Radial Basis Function, and Ladas Super Formula): Which One Is More Accurate?

**DOI:** 10.3390/jcm14072443

**Published:** 2025-04-03

**Authors:** Ionela-Iasmina Yasar, Servet Yasar, Leila Al Barri, Diana-Maria Darabus, Andreea-Talida Tîrziu, Mihnea Munteanu, Horia Tudor Stanca

**Affiliations:** 1 Ophthalmology Department, “Victor Babes” University of Medicine and Pharmacy, 300041 Timisoara, Romania; ionela.yasar@umft.ro (I.-I.Y.); leila.albarri@umft.ro (L.A.B.); diana.darabus@umft.ro (D.-M.D.); andreea.tirziu@umft.ro (A.-T.T.); 2 Munteanu Ophthalmologic Center, 300092 Timisoara, Romania; drservetyasar@gmail.com; 3 Oftalmo Sensory-Tumor Research Center—ORL (EYE-ENT), Ophthalmology Department, “Victor Babes” University of Medicine and Pharmacy, 300041 Timisoara, Romania; 4 Ophthalmology Department, “Carol Davila” University of Medicine and Pharmacy, 050474 Bucharest, Romania; horia.stanca@umfcd.ro

**Keywords:** intraocular lens power calculation formulas, Kane, Barrett universal II, Hill–radial basis function, Ladas super formula, spherical equivalent

## Abstract

**Background:** The most widely used contemporary intraocular lens power calculation formulas are the Kane formula, Barrett Universal II formula, Hill–Radial Basis Function, and Ladas Super Formula, each of which was developed to improve postoperative refractive accuracy. This study aims to conduct a comprehensive comparative analysis of these formulas to evaluate their predictive accuracy across diverse biometric profiles. **Methods:** A total of 210 eyes that met the inclusion criteria were analyzed in this study. This study was designed as a retrospective observational investigation. The biometric parameters of the intraocular lens were evaluated using the ARGOS optical biometer. Refractive intraocular lens power calculations were performed using the formulas, and the resulting values were systematically compared to assess predictive accuracy. In our research, a parametric approach was adopted by applying ANOVA repeated measures analysis. Multiple measurements were evaluated through homogeneity of covariances. Pairwise comparisons between formula-derived values were conducted using the Bonferroni test to identify significant differences. A paired-sample *t*-test was used to compare the spherical equivalent levels calculated at the first and last controls. Potential correlations were examined using Pearson correlation analysis. **Results:** A statistically significant difference was observed between formulas. The differences among the formulas were caused by the values obtained from the Ladas Super Formula being significantly higher than the others. There was a statistically significant positive correlation between the data obtained from the formulas. The spheric equivalent values were similar, with no statistically significant difference. **Conclusions:** This study reinforces the notion that modern intraocular lens power calculation formulas exhibit a high degree of accuracy and correlation in predicting postoperative refractive outcomes.

## 1. Introduction

The accuracy of intraocular lens (IOL) power calculation formulas is fundamental to achieving optimal refractive outcomes in cataracts [1]. Advances in biometric measurements and computational modeling have led to the continuous refinement of these formulas, enhancing their predictive precision [2]. Among the most widely used contemporary formulas are the Kane formula, Barrett Universal II formula (BU II), Hill–Radial Basis Function (Hill–RBF), and Ladas Super Formula (LSF), each developed to improve postoperative refractive accuracy, particularly in eyes with atypical biometric characteristics. Despite their widespread clinical application, ongoing research seeks to determine their relative efficacy across different biometric profiles [3].

The Kane formula, a modern IOL power calculation method, integrates artificial intelligence (AI) with theoretical optics to optimize predictive performance. By leveraging extensive biometric datasets and employing machine learning algorithms, it refines estimations across a broad range of axial lengths and keratometry readings, improving refractive accuracy [4]. Similarly, the BU II formula is grounded in a theoretical model incorporating practical lens position estimation and posterior corneal curvature, making it exceptionally reliable across diverse patient populations [5].

In contrast, the Hill–RBF formula employs a purely data-driven approach, utilizing radial basis function neural networks to analyze complex biometric patterns without relying on predefined mathematical equations. This adaptive methodology allows for continuous self-improvement as additional patient data become available, enhancing its predictive reliability [6]. Finally, the LSF represents a hybrid approach, integrating multiple existing formulas within an algorithmic framework to generate a universal predictive model that dynamically adjusts to individual ocular characteristics [7].

This study aims to conduct a comprehensive comparative analysis of the Kane formula, BU II formula, Hill–RBF, and LSF to evaluate their predictive accuracy across diverse biometric profiles. By assessing their performance in various clinical scenarios, this research seeks to provide ophthalmic surgeons with evidence-based insights, facilitating the selection of the most suitable formula for individual patients. Enhancing IOL power calculation precision may significantly improve surgical outcomes and patient satisfaction.

## 2. Materials and Methods

### 2.1. Study Population

The eyes included in this study were evaluated according to various criteria, and 210 eyes that met these criteria were included. The total number of participants in the study was 175. Of the participants, 101 were female and 74 were male. Although not all participants had both eyes in the study, 35 had both eyes.

### 2.2. Study Design and Participants

This study was designed as a retrospective observational investigation to analyze refractive outcomes following successful IOL implantation. The research was conducted at the Department of Ophthalmology, Victor Babeș University of Medicine and Pharmacy, Timișoara, Romania, between July 2021 and June 2024. Before surgical intervention, all participants underwent a comprehensive ophthalmologic evaluation to assess baseline refractive status and ocular health. The study population included individuals who had undergone IOL implantation to correct refractive errors or cataracts. To ensure measurement consistency, the same examiner conducted all postoperative refractive assessments during a single session, with three consecutive readings obtained and averaged for analysis. The biometric parameters of the IOL were evaluated using the ARGOS optical biometer (Alcon; Forth Worth, TX, USA). Refractive power calculations were performed using the Kane formula, BU II formula, Hill–RBF, and LSF, and the resulting values were systematically compared to assess predictive accuracy. The most commonly preferred formulas for calculating IOL power for myopic [8] and hyperopic [3] eyes, BU II and Kane, were included in the study. The relatively new and promising formulas Hill–RBF and LSF were included to be compared with them. Postoperative follow-up examinations were scheduled at two distinct time points: an early evaluation between 1 and 3 months and a later assessment between 3 and 12 months. During these visits, the spherical equivalent (SE) was calculated. Patients who did not adhere to the scheduled follow-up visits within the designated time frames were excluded from the final analysis.

### 2.3. Power Analysis

The sample size for this study was determined through a power analysis. With an effect size of d = 0.75, power (1 − β) = 0.90, and allocation ratio of 1, the minimum sample size was calculated to be equal in each group, with a minimum of 93 eyes required.

### 2.4. Exclusion Criteria

- History of ocular trauma.
- Previous ocular surgeries.
- AL measurements < 21 mm or >26 mm.
- Corneal or vitreous opacities.
- Dry eye syndrome.
- Retinal pathologies.
- Glaucoma, or nystagmus.
- Participants who did not return for follow-up.

### 2.5. Surgical Procedure

The IOLs used were selected to have the SE value closest to zero according to BU II. The surgical procedure performed on the eyes involved a main incision made at an angle of 110 degrees, with an incision width of 2.2 mm. The first and second side incisions were created at angles of 30 degrees and 160 degrees, respectively, each having an incision width of 1.2 mm. The study utilized monofocal, Extended Depth of Focus (EDOF), and trifocal lenses. The most commonly used brands were Alcon (Alcon Laboratories, Inc., Fort Worth, TX, USA), ZEISS (Carl Zeiss Meditec, Jena, Germany), and Johnson & Johnson (New Brunswick, NJ, USA).

### 2.6. ARGOS

The ARGOS Swept-Source Optical Coherence Tomography (SS-OCT) device (DRI Triton OCT, Topcon, Tokyo, Japan) is a swept-source optical coherence tomography system that operates at a wavelength of 1060 nm, with a bandwidth of 20 nm and an A-scan rate of 3000 scans per second. It provides two-dimensional OCT imaging capabilities. Keratometry (K) was measured using a ring of 16 LEDs with a diameter of 2.2 mm. The reflected image from the LED ring, in combination with the OCT signal, provided accurate corneal curvature results. The device employed a corneal refractive index of 1.3375. The corneal diameter was measured based on the OCT image, which was further utilized as a reference to calculate the white-to-white (WTW) value under the guidance of Alcon’s imaging system. Anterior chamber depth (ACD), Lens Thickness (LT), and axial length (AL) were measured using OCT, taking into account distinct refractive indices for each medium: cornea (1.376), aqueous and vitreous humor (1.336), and lens (1.410).

### 2.7. Examined Variables

- Gender;
- Age of the participants;
- AL;
- ACD;
- LT;
- WTW;
- K (flat and steep);
- SE.

### 2.8. Groups

- Group 1: The Kane formula used in this study was accessed from the latest version of the website.
- Group 2: The Hill–RBF formula used in this study was accessed from the latest version of the website.
- Group 3: The most current version of the BU II formula registered in the Argos device was used on the scheduled date of surgery.
- Group 4: The LSF used in this study was accessed from the latest version of the website [9].

### 2.9. Ethics

The ethical approval for this study was obtained from the Ethics Committee of the Victor Babes University of Medicine and Pharmacy in Timisoara, Romania. All stages of the study were conducted in accordance with the principles outlined in the Declaration of Helsinki.

### 2.10. Statistical Analysis

Statistical analyses were conducted using SPSS version 25 (IBM Corp., Armonk, NY, USA). Descriptive statistics included count, percentage, mean ± standard deviation, median, minimum, and maximum values. The normality of the data was assessed using the Kolmogorov–Smirnov and Shapiro–Wilk tests. Given that the dataset employed in our investigation conformed to a normal distribution, a parametric approach was adopted by applying an ANOVA repeated measures analysis. A sphericity analysis was conducted to assess the homogeneity of covariances across multiple measurements; in instances where covariance equality was not observed, the Greenhouse–Geisser correction was subsequently implemented. Moreover, the origins of the statistical differences among the evaluated formulas were examined using the Bonferroni test. A paired-sample *t*-test was used to compare the SE levels calculated at the first and last controls of the participants. Potential correlations were examined using Pearson correlation analysis. A *p*-value of less than 0.05 was considered the threshold for statistical significance.

## 3. Results

In our study, 210 eyes were examined. The mean age of the participants was 63.44 ± 11.62 years. The oldest participant was 91, while the youngest was 39. The demographic data of the participants and measurement results obtained from ARGOS are presented in Table 1.

In this study, ALCON and ZEISS were the most commonly used IOL brands. Among the ALCON lenses, the subtypes included monofocal, EDOF, and trifocal lenses, with monofocal lenses being the most frequently utilized. Similarly, monofocal and trifocal subtypes were employed for ZEISS lenses, with monofocal lenses being more commonly used than trifocal (Table 2).

**Figure 1:**
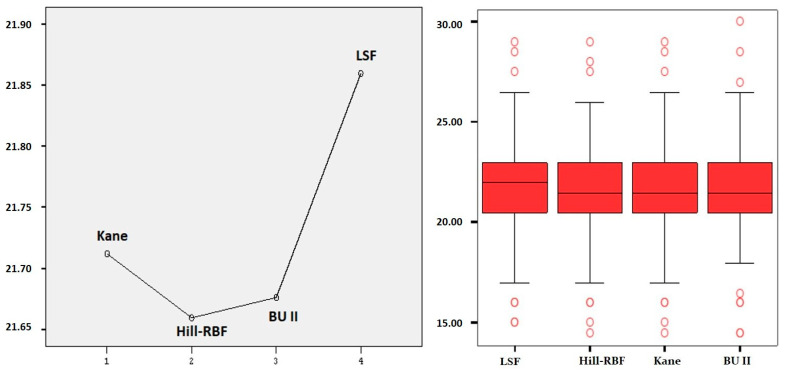
Estimated marginal means and boxplot graphics of formulas.

The lens powers calculated using the Kane formula, Hill–RBF, BU II formula, and LSF were compared. A statistically significant difference was observed between them. Mauchly’s Test of Sphericity was conducted to assess the homogeneity of covariances among multiple measurements. The results indicated that the covariances were not equal. Consequently, the Greenhouse–Geisser correction was applied, revealing that the differences between the formulas were statistically significant. A Bonferroni correction analysis was performed to identify the source of the difference. The analysis using the Bonferroni correction indicates differences in lens power calculations among the formulas because the values obtained from the LSF are significantly higher than those from the other formulas. This difference is statistically significant (Table 3, Figure 1).

Potential correlations between formulas were analyzed using Pearson correlation analysis. The results showed a statistically significant positive correlation between the data obtained from the formulas (Table 4).

We compared the SE values calculated during the participants’ first and second postoperative follow-ups. A paired-sample *t*-test was used to compare the SE levels. The SE values were similar, with no statistically significant difference (Table 5).

**Figure 2:**
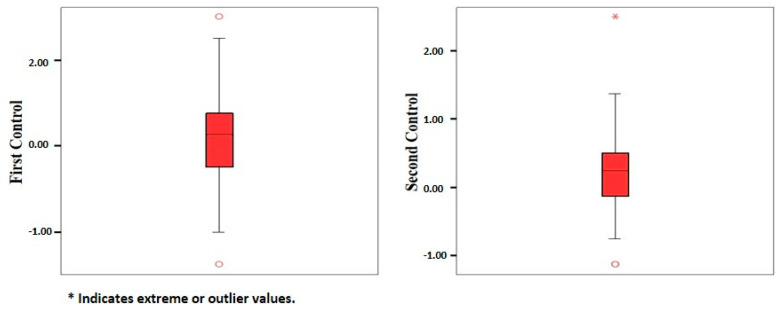
SE values in boxplot graphic.

The analysis of the calculated SE values for both controls revealed that the frequencies of eyes falling within ±1.00 and ±0.50 diopters were similar (Table 6, Figure 2).

## 4. Discussion

Accurately calculating IOL power is paramount to achieving optimal refractive outcomes in cataract surgery. This study systematically compared four widely utilized IOL power calculation formulas—Kane, BU II, Hill–RBF, and LSF—by evaluating their predictive accuracy based on biometric parameters measured using the ARGOS optical biometer. The results underscore important variations among these formulas while demonstrating consistency in refractive predictability.

Our findings indicate a statistically significant difference in the calculated refractive outcomes across the four formulas, suggesting inherent disparities in their computational methodologies. Notably, the LSF yielded significantly higher lens power values than the other three. This discrepancy may be attributed to the LSF’s unique algorithm, which integrates multiple regression models and artificial intelligence-driven predictions. This does not mean that the LSF is less reliable. It can be useful in certain clinical scenarios [10,11]. While the LSF aims to enhance precision by combining multiple IOL formulas, its higher power estimations suggest a potential for overcorrection in some instances [7,12].

The Kane formula, BU II formula, and Hill–RBF produced relatively comparable lens power values, with no significant variations in their predicted refractive outcomes. These formulas incorporate modern artificial intelligence and sophisticated biometric modeling, making them more adaptable to diverse ocular anatomies [13]. In contrast, the LSF’s deviation may indicate its sensitivity to outliers or specific biometric variations, warranting further investigation into its applicability in different patient populations [7,9].

Our results align with recent literature evaluating the performance of contemporary IOL calculation methods. Stopyra et al. investigated seven AI-based formulas, including Hill–RBF 3.0, Kane, PEARL-DGS, LSF AI, Hoffer QST, Karmona, and Zhu-Lu. The LSF approach exhibited more significant prediction variability than the Kane and Barrett Universal II formulas, demonstrating superior predictive accuracy, especially in extremely long eyes. This variability may indicate the LSF’s sensitivity to outliers or specific biometric variations, warranting further investigation into its applicability across different patient populations [11]. These findings reinforce our observation that the LSF systematically generates higher IOL power values, which may introduce a predisposition toward hyperopic outcomes if not interpreted cautiously. The LSF consistently produced significantly higher lens power values than the other formulas. This raises concerns about its potential for overcorrection in some instances, suggesting that the LSF may require further refinement or recalibration based on newer datasets to improve their accuracy.

One of the critical aspects of this study was the postoperative assessment of SE, conducted at two time points (the early postoperative period at 1–3 months and the late postoperative period at 3–12 months). No statistically significant difference was observed in SE values, suggesting that real-world refractive outcomes are ultimately comparable.

Moreover, our analysis revealed a statistically significant positive correlation between the data obtained from all four formulas, indicating a degree of consistency in their computational approach. This aligns with findings from previous studies, such as the work by Melles et al., which demonstrated that while formula-derived IOL power values may differ slightly, their final refractive outcomes tend to converge postoperatively due to biological and healing factors in the eye [14].

Several comparative studies have examined the accuracy of these formulas in predicting postoperative refractive outcomes. Kane et al. reported that the Kane formula exhibited superior accuracy in various biometric ranges, particularly in eyes with shorter and longer axial lengths [15]. Similarly, the Hill–RBF has been praised for its machine learning approach, which has continuously refined its accuracy over time [16]. However, BU II remains widely regarded for its consistency across a broad spectrum of biometric profiles [17].

In contrast, the LSF has been relatively less studied than the other formulas. Some research suggests that its aggregated approach does not consistently outperform standalone formulas, indicating a need for further investigation into its effectiveness and reliability [18,19]. The fact that the LSF yielded significantly higher lens power values in this study suggests that it may require further refinement or recalibration based on newer datasets. The study may require further refinement or recalibration based on newer datasets. This suggests that future research should focus on improving the algorithm to enhance its predictive accuracy and reliability across diverse patient populations [20,21]. A key implication of our findings is the potential for clinical bias. Clinical bias is risky when selecting an IOL power formula, mainly if surgeons rely solely on the LSF without recognizing its tendency to generate higher values. This could lead to the overestimation of IOL power and subsequent postoperative hyperopic shifts [22,23]. This observation is crucial in light of recent advancements in AI-driven formulas, which continue to refine their accuracy based on increasingly expansive datasets. There is a need for more extensive comparative studies involving the LSF and other IOL power calculation formulas. Such studies could help clarify the conditions under which the LSF performs optimally and identify specific patient profiles that may benefit from its use.

## 5. Conclusions

This study reinforces the notion that modern IOL calculation formulas exhibit high accuracy and correlation in predicting postoperative refractive outcomes. While differences exist in calculated IOL power, particularly with the LSF producing higher values, the final spherical equivalent outcomes remain statistically similar across all formulas. Despite differences in calculated IOL power values, real-world refractive outcomes remain comparable among the formulas.

Our study did not perform subgroup analyses that could affect formula performance, such as axial length and corneal parameters. This suggests that different results may be observed in cases of high myopia or short axial length.

Ultimately, the IOL calculation formula should be tailored to individual patient characteristics, emphasizing optimizing biometric inputs to achieve the most precise refractive outcomes. Further research with larger sample sizes and subgroup analyses based on axial length and corneal parameters would provide deeper insights into the nuanced performance of these formulas.

Conducting longitudinal studies to assess the long-term outcomes of patients using different IOL power calculation formulas could provide valuable insights. This would help determine the real-world effectiveness of these formulas in achieving desired refractive outcomes over time.

## Figures and Tables

**Table 1:** Descriptive statistics.

	*n*	Min	Max	Mean ± SD
Age	210	39	91	63.44 ± 11.62
AL		21.13	25.77	23.2818 ± 0.83
K1		39.99	48.33	43.5660 ± 1.45
K2		40.50	48.89	44.2356 ± 1.52
ACD		2.52	4.11	3.2176 ± 0.33
LT		3.45	5.53	4.5334 ± 0.37
WTW		10.80	12.93	11.9417 ± 0.37

**Table 2:** Descriptive statistics of the two most commonly used lens brands and their subtypes classified by focal points.

	*n*	%
ALCON *	129	61.1
Monofocal **	58	44.9
EDOF **	23	17.8
Trifocal **	48	37.3
ZEISS *	44	20.8
Monofocal **	28	36.6
Multifocal **	16	63.4

* The frequency distribution of all brands utilized in the study. ** The frequency distribution within the ALCON/ZEISS brand.

**Table 3:** Comparison of Kane formula, Hill–RBF, BU II formula, and LSF in terms of lens power.

		Mean ± SD		
Kane		21.71 ± 2.16		
Hill–RBF		21.65 ± 2.10		
BU—II		21.68 ± 2.11		
LSF		21.85 ± 2.12		
Within-Subjects Effect	Mauchly’s W	Approx. Chi-Square	df	Sig.
Formula	0.936	13.759	5	0.017
Greenhouse–Geisser	df	Mean Square	F	Sig.
	2.88	1.81	37.14	0.000 (4 > 3, 2, 1)

**Table 4:** Correlation analysis between BU II and LSF.

		Kane	Hill–RBF	BU-II	LSF
Kane	Pearson Correlation	1	0.991 (**)	0.988 (**)	0.990 (**)
	Sig. (2-tailed)		0.000	0.000	0.000
Hill_RBF	Pearson Correlation	0.991 (**)	1	0.991 (**)	0.991 (**)
	Sig. (2-tailed)	0.000		0.000	0.000
BU II	Pearson Correlation	0.988 (**)	0.991 (**)	1	0.988 (**)
	Sig. (2-tailed)	0.000	0.000		0.000
LSF	Pearson Correlation	0.990 (**)	0.991 (**)	0.988 (**)	1
	Sig. (2-tailed)	0.000	0.000	0.000	

** Correlation is significant at the 0.01 level (2-tailed).

**Table 5:** Comparison of SE values calculated during the first and second postoperative follow-ups.

	*n*	Mean ± SD	Std. Error	t	df	*p*
First Control	210	0.13 ± 0.48	0.03	−0.4	418	0.6
Second Control	210	0.14 ± 0.49	0.03			

**Table 6:** Frequencies of the eyes within ±1.00 diopters and ±0.50 diopters.

	*n*	±1 D	±0.50 D
First Control	210	%96.7	%89.6
Second Control	210	%96.2	%89.6

## Data Availability

The data supporting the findings of this study are available from the corresponding author upon reasonable request.

## Abbreviations

AI	Artificial Intelligence
IOL	Intraocular Lens
AL	Axial Length
ACD	Anterior Chamber Depth
SS-OCT	Swept-Source Optical Coherence Tomography
BU II	Barrett Universal II
HILL-RBF	Hill–Radial Basis Function
LSF	Ladas Super Formula
SE	Spherical Equivalent
EDOF	Extended Depth of Focus
WTW	White-to-White
CCT	Central Corneal Thickness
K	Keratometry
